# Cross-Scale Guidance Integration Transformer for Instance Segmentation in Pathology Images

**DOI:** 10.1109/OJEMB.2025.3555818

**Published:** 2025-03-28

**Authors:** Yung-Ming Kuo, Jia-Chun Sheng, Chen-Hsuan Lo, You-Jie Wu, Chun-Rong Huang

**Affiliations:** Department of Electronic EngineeringNational Formosa University 63370 Yunlin County 632 Taiwan; Department of Computer Science and EngineeringNational Chung Hsing University 34916 Taichung 402 Taiwan; Department of Computer ScienceNational Yang Ming Chiao Tung University 34914 Hsinchu 300 Taiwan

**Keywords:** Instance segmentation, pathology image, self-attention, transformer

## Abstract

*Goal:* To assess the degree of adenocarcinoma, pathologists need to manually review pathology images. To reduce their burdens and achieve good inter-observer as well as intra-observer reproducibility, instance segmentation methods can help pathologists quantify shapes of gland cells and provide an automatic solution for computer-assisted grading of adenocarcinoma. However, segmenting individual gland cells of different sizes remains a difficult challenge in computer aided diagnosis. *Method:* A novel cross-scale guidance integration transformer is proposed for gland cell instance segmentation. Our network contains a cross-scale guidance integration module to integrate multi-scale features learned from the pathology image. By using the integrated features from different field-of-views, the decoder with mask attention can better segment individual gland cells. *Results:* Compared with recent task-specific deep learning methods, our method can achieve state-of-the-art performance in two public gland cell datasets. *Conclusions:* By imposing cross-scale encoder information, our method can retrieve accurate gland cell segmentation to assist the pathologists for computer-assisted grading of adenocarcinoma.

## I. Introduction

The assessment of the malignant degree of adenocarcinoma and treatment planning are highly dependent on the glandular morphology analysis in pathology images. In routine pathological practice, pathologists must manually review pathology images of patients for evaluation. Accurate segmentation of gland cells is crucial because cell morphological characteristics can help pathologists assess the malignant degree of adenocarcinoma more accurately [1]. In particular, automated gland segmentation in pathology images allows pathologists to extract essential morphological features from large-scale pathology images. Therefore, to avoid the burdens of pathologists and increase the accuracy, efficiency, and consistency of the diagnosis, artificial intelligence (AI)-based instance segmentation of gland cells of variant sizes is required. In early approaches [2], [3], [4], [5], [6], [7], glandular structure segmentation relied on hand-crafted features such as texture and morphological properties extracted from pathology images. Nevertheless, these hand-crafted features also restrain the performance of these methods.

In comparison, deep learning methods such as convolutional neural network (CNN)-based methods have been shown to be more effective in solving image classification problems [8], [9], [10], [11], [12]. They are also applied to solve pathology image classification problems [13], [14], [15], [16] or semantic segmentation problems [17], [18]. To further segment individual cells for the glandular morphology analysis of pathology images, CNN-based instance segmentation methods are proposed [19], [20], [21], [22], [23], [24], [25], [26]. However, the field-of-views of these CNN-based methods are restricted by the kernel sizes, which may lead to the problems of segmenting gland cells of variant sizes. In addition, convolutional filters provide local information, which is difficult to consider global information on the pathology image. Thus, attention networks and Gabor texture features at different scales are considered in [27], and topology information by using gland cell skeletons and markers is considered in [28] to improve network performance.

In recent years, transformer-based methods [29], [30], [31], [32], [33] have been shown to be effective in solving computer vision problems and medical image processing problems [11] by considering self-attention schemes and are applied to pathology image classification [15], [16]. To investigate the potential of the transformer for instance segmentation in pathology images, Sheng et al. [34] study the performance of Mask2Former [35] in pathology datasets [22], [36]. Although Mask2Former aims to achieve instance segmentation in general computer vision tasks, it achieves comparable performance in instance segmentation of pathology image datasets. The scale of the encoder in Mask2Former is the same for the features generated by each stage and may lead to the difficulty of segmenting gland cells of variant sizes. Nevertheless, such an investigation encourages the development of more effective transformer-based methods for instance segmentation in pathology images.

**Fig. 1.**
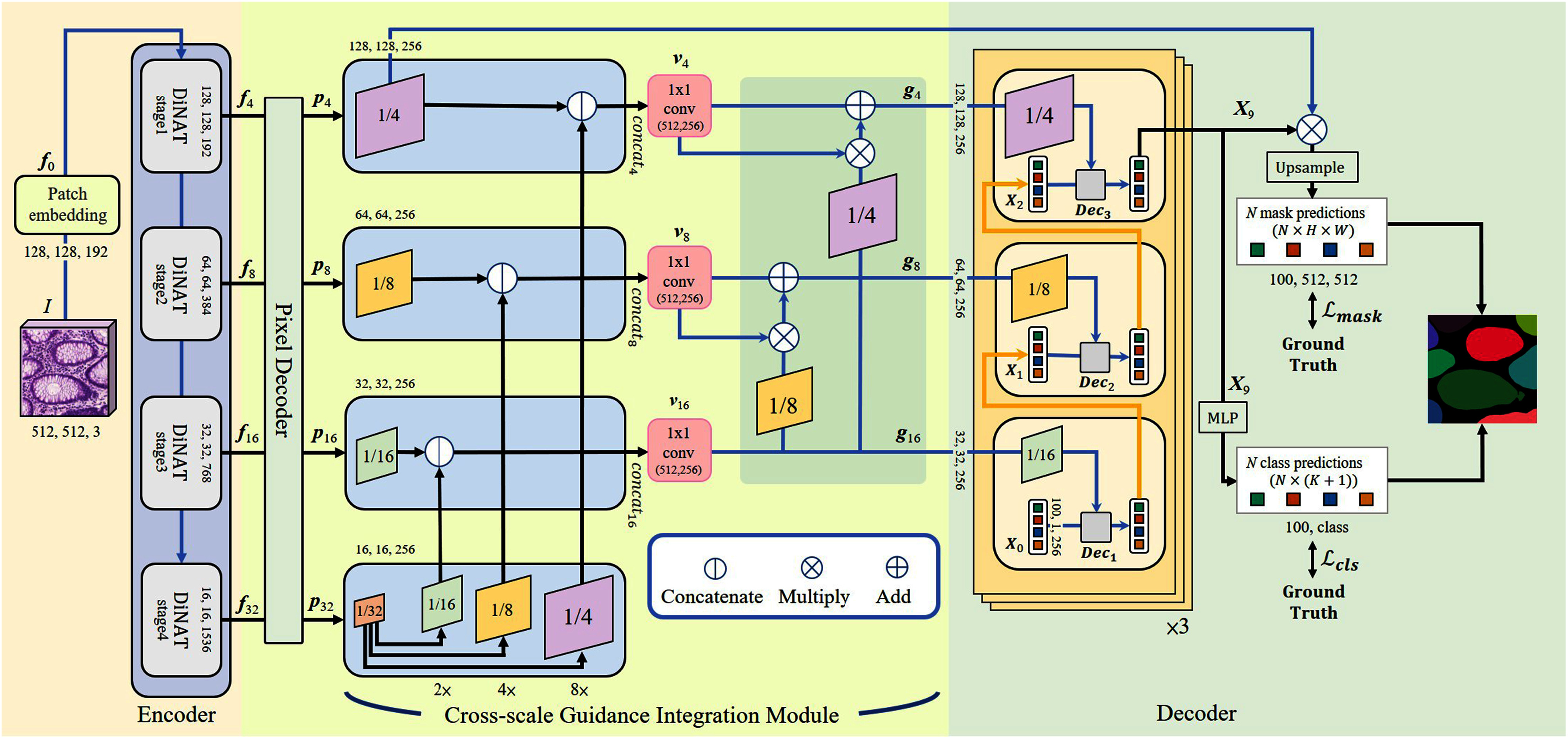
The network architecture of the cross-scale guidance integration transformer. It contains an encoder backbone, a cross-scale guidance integration module, and a transformer decoder. The cross-scale guidance integration module integrates features with the larger field-of-views to guide features with the smaller field-of-views to increase the feature representation ability at each layer. The integrated features help the transformer decoder produce better instance segmentation results and help the network learn more efficiently. $\bm {f}_{0}$ is the patch embedding. $\bm {f}_{4}$, $\bm {f}_{8}$, $\bm {f}_{16}$, and $\bm {f}_{32}$ are the encoder features generated by the stage 1, stage 2, stage 3, and stage 4 of the dilated neighborhood attention transformer, respectively. $\bm {p}_{4}$, $\bm {p}_{8}$, $\bm {p}_{16}$, and $\bm {p}_{32}$ are the decoder features generated by different scales of the pixel decoder. $\bm {v}_{4}$, $\bm {v}_{8}$, and $\bm {v}_{16}$ are the output features of the first block in the cross-scale guidance integration module. $\bm {g}_{4}$, $\bm {g}_{8}$, and $\bm {g}_{16}$ are the output features of the second block in the cross-scale guidance integration module. $\bm {X}_{l}$ represents the feature of the $l$th transformer decoder.

To address the variant size problem of gland cell instance segmentation, we propose a novel cross-scale guidance integration transformer. Fig. 1 shows three main parts of our model, including an encoder backbone for feature extraction, a cross-scale guidance integration module to integrate cross-scale information to assist the learning of the transformer decoder, and a transformer decoder for instance segmentation. In the encoder, we employ a dilated neighborhood attention transformer [33] with four stages to obtain multi-scale features from the pathology image. To generate a better feature representation for the transformer decoder, a cross-scale guidance integration module is presented. It upsamples features with the largest field-of-view in the fourth stage of the encoder and integrates them with the features learned in the first three stages. In this way, we can use the features with the largest field-of-view to assist in training features with smaller field-of-views.

Moreover, we also integrate features of different scales to learn cross-scale features for the transformer decoder based on the features of the first three stages. The transformer decoder can then receive more information from different field-of-views to segment gland cells of variant sizes. Finally, the feature of the first stage of the encoder is collaborated with the transformer decoder to obtain individual gland cells. The model is trained based on the mask loss and the classification loss [35]. Experiments are conducted on the CRAG [22] and GlaS [36] datasets. Visualizations on segmented gland cells reveal that the results of instance segmentation of cells and the depictions of cell edges of the proposed method are also more accurate compared with competing methods. Our main contributions are as follows.

• First, a cross-scale guidance integration module is proposed to use the features with the largest field-of-view to guide the learning of features with the smaller field-of-views for better feature representation.
• Second, features of different scales are combined to cross-scale features for the transformer decoder to segment gland cells of variant sizes.
• Third, the proposed method can achieve the state-of-the-art performance in public datasets compared with competing methods.

The paper is organized as follows. Section II gives the materials and methods. The results are revealed in Section III. Finally, conclusions are given in Section IV.

## II. Materials and Methods

### A. Overview

Given a pathology image $I$ with size $H \times W \times C$, where $H$, $W$, and $C$ are the height, width, and channel of $I$, an initial downsampler [33] is used to generate the patch embedding $\bm {f}_{0}$ of a spatial size $1/4$ of the size of the pathology image. Fig. 1 reveals the network architecture. To generate features with different scales, a dilated neighborhood attention transformer [33] with four stages is used as the encoder backbone. After each stage, the spatial size is downsampled by $1/2$ and we double the number of channels. Thus, the dimensions of the features $\bm {f}_{4}$, $\bm {f}_{8}$, $\bm {f}_{16}$, and $\bm {f}_{32}$ of the four stages are $1/4$, $1/8$, $1/16$, and $1/32$ of the input image size, respectively.

Although the dimension of the features $\bm {f}_{32}$ in the fourth stage is smaller, these features have the largest field-of-view. Therefore, we apply the learned features of the fourth stage, which give the global views of the pathology image to assist in learning features in the first three stages during training. Based on the above observations, we develop a cross-scale guidance integration module to help the instance segmentation network learn rich cross-scale features. Finally, the transformer decoder is applied to impose cross-scale features of each scale to generate a segmentation map whose scale is $1/4$ of the original image size. The segmentation map is used to compute mask loss $\mathcal {L}_{mask}$ and classification loss $\mathcal {L}_{cls}$ for training the network.

### B. Cross-Scale Guidance Integration Transformer

#### 1) Encoder Backbone

In the vision transformer [29], it is required to perform self-attention on all patches, which is time consuming for computation, as indicated in [31]. To further reduce the computation of self-attention computation and consider spatial neighbor correlations between gland cells in the pathology image, we propose using the dilated neighborhood attention transformer [33], which has been shown to capture a more global context by expanding the field-of-view of features. The dilated neighborhood attention transformer (DiNAT) initially reduces the input to $1/4$ of its original spatial resolution and sends it through the 4 stages of the encoders. The feature map is reduced to half its spatial size and doubled in the channels between stages. Please refer to [33] for detailed implementation.

A multi-scale deformable attention transformer [37] is applied as a pixel decoder. Each layer of the multi-scale deformable attention transformer applies to feature maps with $1/8$, $1/16$, and $1/32$ of the input image size and uses an upsampling layer with a lateral connection on the feature map of $1/8$ of the input image size to generate the feature map of $1/4$ of the input image size. Please refer to [37] for detailed implementation. The encoder features $\bm {f}_{4}$, $\bm {f}_{8}$, $\bm {f}_{16}$, and $\bm {f}_{32}$ are sent to the pixel decoder to obtain multi-scale features $\bm {p}_{4}$, $\bm {p}_{8}$, $\bm {p}_{16}$, and $\bm {p}_{32}$. However, the observed scales of the learned features are independent. To further represent gland cells of variant sizes by considering features of different scales, the cross-scale guidance integration module is proposed in the following.

#### 2) Cross-Scale Guidance Integration Module

To simultaneously consider multiple field-of-view information, we propose the cross-scale guidance integration module. The module consists of two blocks. The first block aims to use the feature $\bm {p}_{32}$ with the largest field-of-view to help train the features $\bm {p}_{4}$, $\bm {p}_{8}$, and $\bm {p}_{16}$ with smaller field-of-views learned in the first three stages. To overcome the dimension inconsistency problem among these features, we use bicubic interpolation to enlarge the feature $\bm {p}_{32}$ by scale factors of 2, 4, and 8, respectively. Then, the upsampled feature by the scale factor $j$ is concatenated with the feature $\bm {p}_{k}$. The features learned in the last stage have the largest field-of-view. By combining features with the largest field-of-view, features with smaller fields-of-views can fuse global information to improve the prediction results.

Although the concatenation provides global information for the features learned in different field-of-views, the number of channels also increases. To maintain dimension with respect to the transformer decoder, the concatenated features are passed to the $1 \times 1$ convolutional layers to make the channels the same as the original learned features, i.e. 256. In order to decrease network parameters, the shared-weights scheme is employed in the $1 \times 1$ convolutional layers that contain 256 $1 \times 1$ convolutional kernels to generate feature maps with 256 channels. The output $\bm {v}_{k}$ of $\bm {p}_{k}$ after the first block is defined as follows:

\begin{equation*}
 {v}_{k} = conv^{1}({U^{j}( {p}_{32})} \bigcirc\; {p}_{k}), \tag{1}
\end{equation*}

where $conv^{1}(\cdot)$ is the $1 \times 1$ convolutional function, $U^{j}(\cdot)$ is the bicubic interpolation function to enlarge the spatial resolution of feature $\bm {p}_{32}$ with the scale factor $j$, ◯ is the concatenation operator, $k = 32/j$, and $j\in \lbrace 2, 4, 8\rbrace$. Then, we send these features to the second block of the proposed module.

In the second block, we further fuse features of different scales to enrich the multi-scale information for boosting the performance of the transformer decoder. We use $\bm {v}_{16}$, which provides representative features of a larger field-of-view compared with $\bm {v}_{8}$ to complement $\bm {v}_{8}$. $\bm {v}_{16}$ is upsampled by the scale factor of 2 and serves as the weight map to multiply $\bm {v}_{8}$. We then add the multiplied results to $\bm {v}_{8}$ to help feature learning by considering cross-feature fusion. Moreover, the added results preserve more detailed information in the smaller field-of-view. Similarly, we also use $\bm {v}_{16}$ by upsampling with the scale factor 4 to complement $\bm {v}_{4}$. Then, the integrated feature $\bm {g}_{k}$ of the second block can be expressed as:

\begin{equation*}
\bm {g}_{k}={U^{j}}(\bm {v}_{16})\otimes \bm {v}_{k} \oplus \bm {v}_{k}, \tag{2}
\end{equation*}

where $\otimes$ is the multiplication operator, $\oplus$ is the addition operator, $k = 16/j$, and $j\in \lbrace 2, 4\rbrace$. Here, $\bm {g}_{16}$ equals $\bm {v}_{16}$. In this way, the integrated features contain information from different field-of-views (scales) to help the decoder better represent gland cells of variant sizes.

#### 3) Transformer Decoder

Instead of using conventional attention layers, we employ the masked-attention scheme [35] to enforce the network focusing on gland cell regions. The masked attention cooperates with the integrated features learned from the cross-scale guidance integration module to retrieve important transformer decoder features to represent glandular tissues of variant sizes.

Given an integrated feature $\bm {g}_{k}$ for the $l$th transformer decoder, two transformation functions $f_{K}(\cdot)$ and $f_{V}(\cdot)$ are used to transfer $\bm {g}_{k}$ to $\bm {K}_{l}\in$ $\mathbb {R}^{{H}_{l}{W}_{l}\times C}$ and $\bm {V}_{l}\in$ $\mathbb {R}^{{H}_{l}{W}_{l}\times C}$ for cross-attention computation, where ${H}_{l}$ and ${W}_{l}$ are the width and height of $\bm {g}_{k}$. The masked-attention [35] is defined as follows:

\begin{equation*}
\bm {X}_{l}=\text{softmax}\left(\mathcal {M}_{l-1}+\bm {Q}_{l} \bm {K}_{l}^{\mathrm{T}}\right) \bm {V}_{l}+\bm {X}_{l-1}, \tag{3}
\end{equation*}

where $l$ is the index of the decoder and $\bm {Q}_{l} = f_{Q}(\bm {X}_{l-1}) \in \mathbb {R}^{N\times C}$ is the query feature of the ${l}$th decoder. Here, $\bm {X}_{0}$ is a learnable input query feature for the first decoder. Then, the zero-initialized query feature $\bm {X}_{0}$ is updated based on the integrated features generated by the previous module. The attention mask $\mathcal {M}_{l-1}$ equals zero if the binary mask prediction of (${l-1}$)th transformer decoder is 1 and equals $-\infty$ if the binary mask prediction is 0. With the binary mask prediction, the current transformer decoder can focus on learning object regions.

The decoder features are sent to the next decoder to further learn representative features based on integrated features of different scales and queries obtained from the previous decoder. This concept is actually the same as the region proposals [38], which represent areas that are likely to be objects. Here, the first mask prediction is obtained from the initialized query features, i.e. before sending query features to the transformer decoder. Then, each transformer decoder will generate a mask prediction and resize it to the scale required by the next transformer decoder layer to calculate the masked attention. Each layer in the transformer decoder, except for the first layer, can help the learning of the next layer.

For example, if the query features find the target cell in the bottom left corner of the feature map of $1/16$ of the size of the input image, it will also tend to find the target cell in the bottom left corner of the feature map of $1/8$ of the size of the input image for consistency. We use the integrated features with the resolutions $1/16$, $1/8$, and $1/4$ of the pathology image to three transformer decoders and repeat the process three times. After the integrated features and query features pass through the first transformer decoder to the last transformer decoder, the output feature $\bm {X}_{9}$ is generated. Then, $\bm {X}_{9}$ is multiplied by $\bm {p}_{4}$ using matrix multiplication to obtain $N$ binary mask predictions, where $N$ is the number of queries. The spatial resolutions of these $N$ binary mask predictions will be $1/4$ of the size of the pathology image. Finally, we upsample the predictions to generate a result image with the same resolution as the pathology image. Moreover, the feature generated by the last transformer decoder, i.e. $\bm {X}_{9}$, is sent to a multilayer perceptron (MLP) classifier to generate category predictions. With the above results, we can generate the segmentation results of gland cells.

## III. Results

### A. Datasets and Experimental Settings

The colorectal adenocarcinoma gland (CRAG) dataset [22] and the gland segmentation (GlaS) dataset [36] are two commonly used datasets for gland segmentation. The CRAG dataset contains 213 colon pathology images in which gland cells are individually labeled. There are 173 training images and 40 testing images in the CRAG dataset, respectively. The resolutions of the images range from $1319 \times 1516$ to $1514 \times 1516$ pixels in the PNG format. All whole slide images are from different patients to provide inter-subject variability of patients. The gland segmentation (GlaS) dataset contains 85 training and 80 testing images. The test images are divided into subgroup A (60 images) and subgroup B (20 images). The resolutions of the images range from $567 \times 430$ to $775 \times 522$ pixels in the BMP format. High inter-subject variability in the stain distribution and tissue architecture leads to the segmentation difficulty of the dataset. The datasets are summarized in Table I. For more details on the datasets, please refer to [22] and [36]. Given the training images, we perform data augmentations [44] as the preprocessing step to avoid overfitting. The data augmentations include randomly cropping, horizontal flip, vertical flip, color jittering and rotations of $90, 180$, and 270 degrees to enrich the training images.

**TABLE I:** Datasets

Dataset	Training	Testing
CRAG [22]	173	40
GlaS [36]	85	60 (A) + 20 (B)

To evaluate performance, three metrics are calculated including the score of the pixel-level metric ${F_{\mathrm{{1}}}}$, the object-level dice index, and the Hausdorff distance, which are the same as the metrics reported in [24], [25], [27]. During inference, given a pathology image, the proposed method will perform instance segmentation for the image and report the number of segmented malignant cells so that pathologists can assess the cancer grades without manually counting the malignant cells.

### B. Quantitative Results

CNN-based instance segmentation methods that include Mask R-CNN [39], CMD-Net [40], TCC-MSF [24], DSF-CNN [25], GCSBA-Net [27], TA-Net [28], DSE [23], DoubleU-Net [26] and FRMDR-Net [43], transformer-based methods that include Co-DETR [42] and Mask2Former [35], and the diffusion model-based method that include IAD [41] are compared. Table II reveals the quantitative results of competing CNN-based methods, diffusion model-based methods, transformer-based methods, and the proposed method for the CRAG dataset. Although Mask2Former is not proposed for pathology image instance segmentation, it can achieve superior Dice and Hausdorff distance results compared with competing methods. Such results indicate the potential of transformer-based methods for segmenting gland cells in pathology images. Compared with Mask2Former and Co-DETR, the proposed method considers the cross-scale information extracted by using the cross-scale guidance integration module to learn representative features of transformer decoders. Thus, the boundaries of gland cells with respect to variant sizes are accurately described by the proposed method, and the proposed method has the best Dice and Hausdorff distance results for the CRAG [22] dataset.

Table III shows the quantitative results of the GlaS dataset. Our method has better results for the testing subset A and comparative results for the testing subset B compared with most competing methods. Moreover, the Hausdorff distance results of the proposed method are better compared with most competing methods, indicating that the segmented boundaries of the proposed method are closer to the boundaries of the ground truth gland cells. Such results can help pathologists achieve more accurate glandular morphology analysis.

**TABLE II:** Comparisons With Competing Methods for the CRAG Dataset

Methods	$F_{1}$	Dice	Hausdorff
Mask R-CNN [39]	0.771	0.799	190.99
CMD-Net [40]	0.840	0.879	132.38
TCC-MSF [24]	0.876	0.892	130.03
DSF-CNN [25]	0.874	0.891	138.40
GCSBA-Net [27]	0.836	0.894	146.77
DSE [23]	0.835	0.889	120.13
DoubleU-Net [26]	0.835	0.890	117.25
IAD [41]	0.853	0.906	113.22
TA-Net [28]	0.842	0.893	105.20
Co-DETR [42]	0.806	0.814	247.63
Mask2Former [35]	0.847	0.899	104.70
Proposed	0.875	0.900	102.11

**TABLE III:** Comparisons With Competing Methods for the GlaS Dataset

Methods	$F_{1}$		Dice		Hausdorff	
	TestA	TestB	TestA	TestB	TestA	TestB
Mask R-CNN [39]	0.779	0.578	0.815	0.679	80.19	174.56
CMD-Net [40]	0.919	0.860	0.912	0.848	40.13	98.32
TCC-MSF [24]	0.914	0.850	0.913	0.858	39.85	93.24
GCSBA-Net [27]	0.916	0.832	0.914	0.834	41.49	102.88
DSE [23]	0.926	0.862	0.927	0.871	31.21	80.51
DoubleU-Net [26]	0.935	0.871	0.929	0.875	27.84	76.05
IAD [41]	0.941	0.893	0.939	0.889	26.04	72.35
FRMDR-Net [43]	0.898	0.817	0.915	0.852	37.27	91.56
Co-DETR [42]	0.883	0.778	0.877	0.781	69.27	121.21
Mask2Former [35]	0.905	0.813	0.901	0.842	48.63	95.06
Proposed	0.919	0.820	0.920	0.851	35.92	87.14

### C. Qualitative Results

**Fig. 2.**
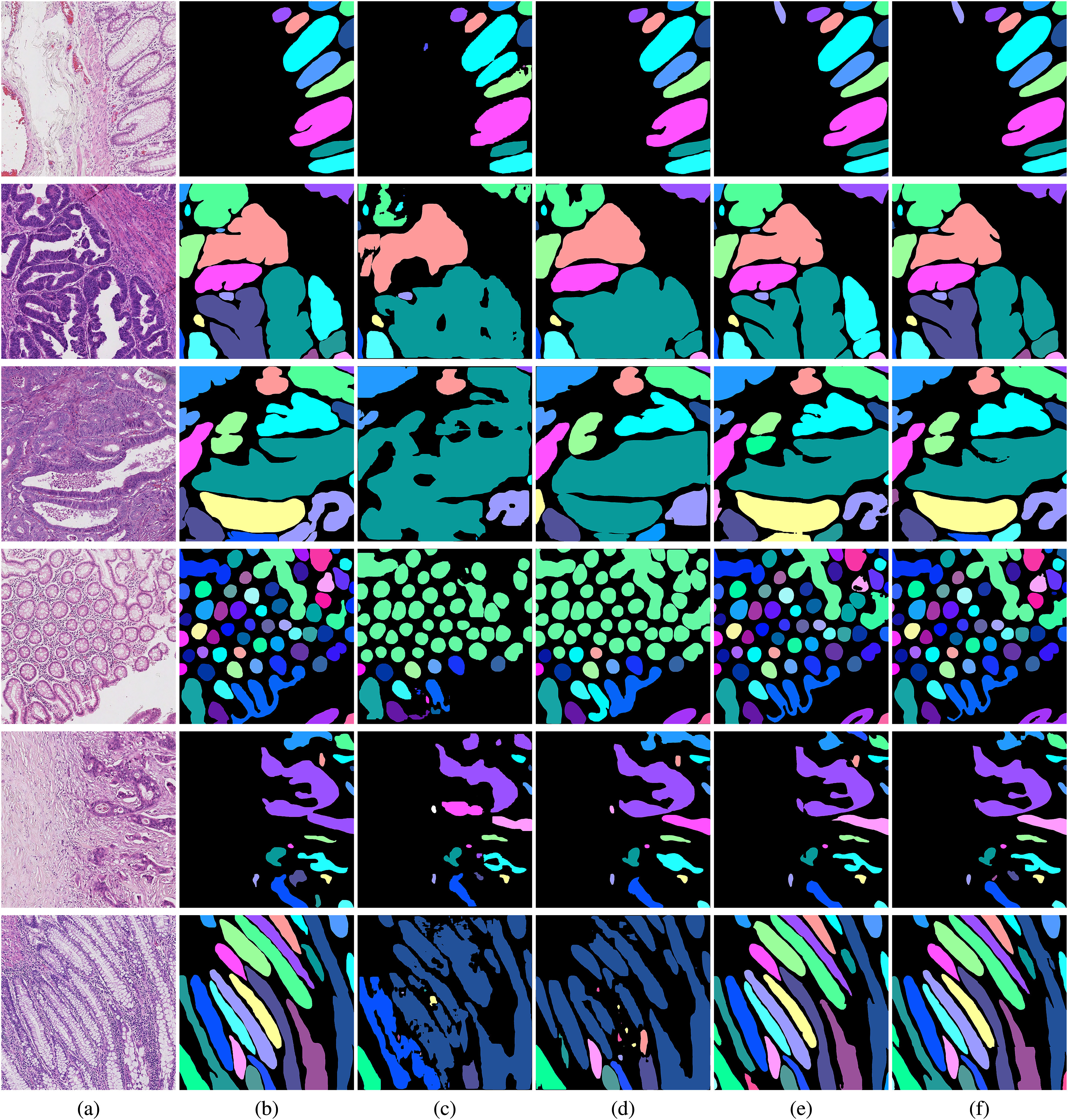
Qualitative results for the CRAG dataset. Regions of different colors represent different gland cells. (a) Original images, (b) Ground truth, (c) Mask-RCNN, (d) Co-DETR, (e) Mask2Former, and (f) Proposed method.

The original pathology images and ground truth instance segmentation labels from the CRAG dataset [22] are shown in Fig. 2(a) and (b), respectively. Regions of different colors represent different gland cells. As shown in each row of Fig. 2(a), the sizes and shapes of gland cells are variant, which leads to the difficulty of instance segmentation. The results of Mask R-CNN [39], Co-DETR [42], Mask2Former [35], and our method are shown in Fig. 2(c), (d), (e), and (f), respectively. Mask R-CNN fails to segment gland cells as shown in Fig. 2(c). Compared with Mask R-CNN, Co-DETR and Mask2Former usually over-segment the gland cells, i.e. merge similar neighboring gland cells. In contrast, by further integrating cross-scale information, our method can segment the gland cells of variant sizes without merging similar neighbor gland cells to the same cell. Furthermore, the shapes of segmented gland cells in the proposed method are closer to the true shapes of the gland cells, which reflect the Hausdorff distance results reported in Table II. Such results help pathologists assess cancer grades by accurately quantifying the shapes of gland cells.

**Fig. 3.**
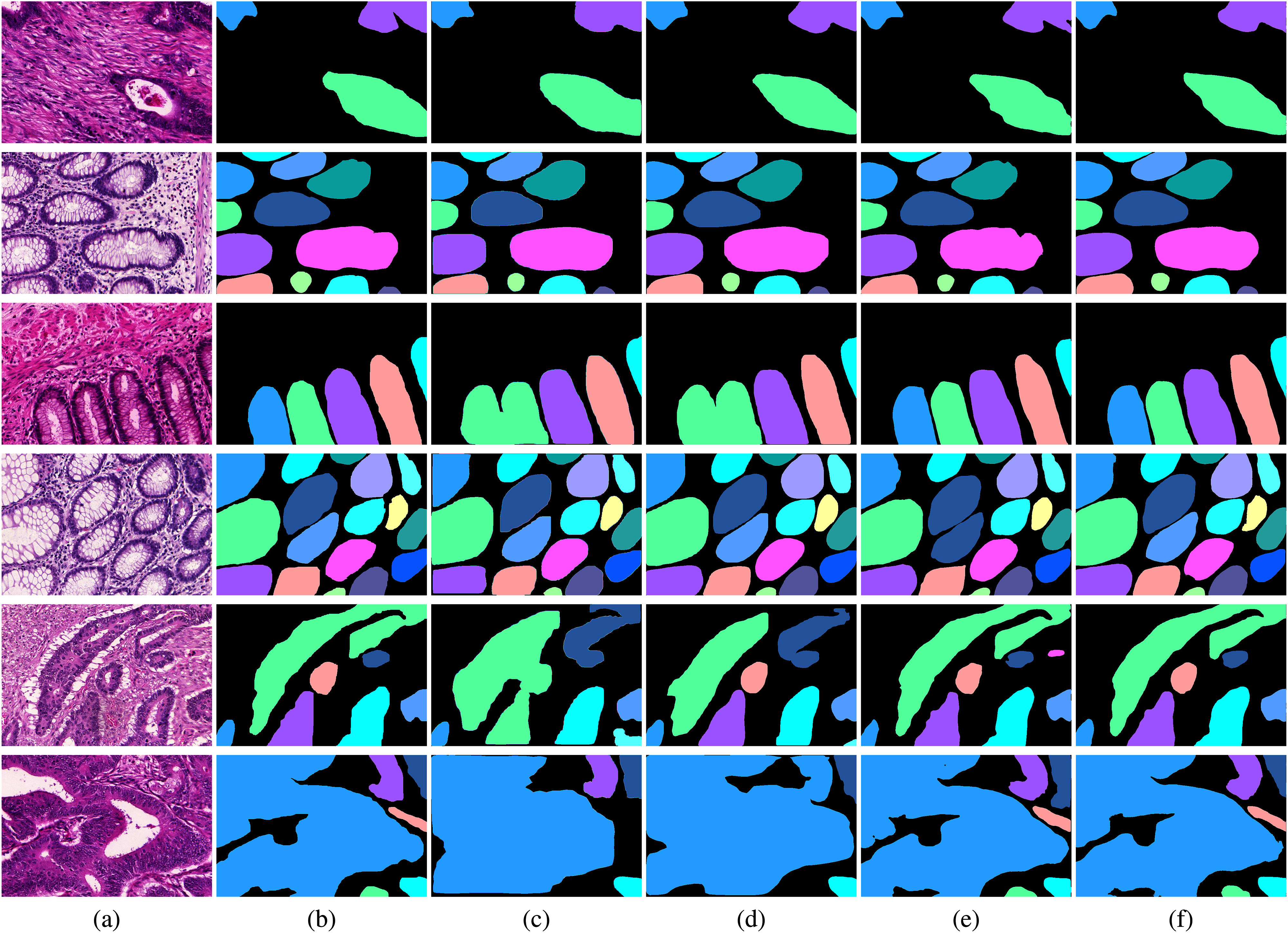
Qualitative results for the GlaS dataset. Regions of different colors represent different gland cells. (a) Original images, (b) Ground truth, (c) Mask-RCNN, (d) Co-DETR, (e) Mask2Former, and (f) Proposed method.

Fig. 3(a) and (b) show the pathology images and ground truth instance segmentation labels of the GlaS [36] dataset. Similar to the results in the CRAG dataset, the instance segmentation results of Mask-RCNN, Co-DETR, and Mask2Former shown in the three, fourth, fifth, and sixth rows of Fig. 3(c), (d), and (e) contain over-segmentation results. Considering cross-scale information for the transformer decoder, our method can have better gland cell segmentation results, as shown in Fig. 3(d). It solves the gland cell instance segmentation problem for glandular morphology analysis of pathology images. The results of instance segmentation can provide pathologists an automatic solution for quantified shapes of gland cells and computer-assisted adenocarcinoma grading. Our method also reduces the burden of pathologists and achieves good inter-observer as well as intra-observer reproducibility for computer-assisted grading of adenocarcinoma in the clinic.

## IV. Conclusion

In summary, we propose a cross-scale guidance integration transformer for gland cell instance segmentation in pathology images. The experimental results reveal that our method can automatically segment gland cells of variant sizes. The segmented cells can be used to help pathologists assess the degree of adenocarcinoma. The results not only reduce the burdens of the pathologists, but also provide good inter-observer reproducibility for computer-assisted grading of adenocarcinoma. In the future, the proposed method will be applied to segment different types of cells, such as tumor cells from liver tissues [17] in pathology images.

## Supplementary Materials

The supplementary materials include details related to the loss functions and implementation details in the Materials and Methods section, and the ablation study in the Results section.

Supplementary Materials

## Conflict Of Interest

The authors have no conflict of interest to declare.

## Author Contributions

Yung-Ming Kuo contributed to the conceptualization, the design of the methodology, and data analysis. Jia-Chun Sheng contributed to the conceptualization, the design of the methodology and the implementation of the network. Chen-Hsuan Lo contributed to the implementation of the network and performed the experimental results and the ablation study. You-Jie Wu performed the experimental results and the ablation study. Chun-Rong Huang contributed to the conceptualization, the design of the methodology, refined the manuscript, and supervised the project. All authors contributed to the discussion of the results and the writing of the manuscript.
