# Supplementary material for: Cross-Scale Guidance Integration Transformer for Instance Segmentation in Pathology Images
Source: IEEE Open J Eng Med Biol. 2025 Mar 28;6:413–9. doi: 10.1109/OJEMB.2025.3555818 (PMC12250935; doi:10.1109/OJEMB.2025.3555818)
Supplement: Supplementary Materials [file supp1-3555818.pdf]

Supplementary Materials

Cross-scale Guidance Integration Transformer for Instance Segmentation in Pathology Images

Yung-Ming Kuo, *Member, IEEE*, Jia-Chun Sheng, Chen-Hsuan Lo, You-Jie Wu, Chun-Rong Huang, *Senior Member, IEEE*

I. Materials and Methods

A. Loss

COMPARED to classification tasks, semantic and instance segmentation tasks require a lot of memory during training. The reason is that the resolution of the pathology image is relatively high. Therefore, the memory requirement is reduced by computing the mask loss of the random sampling points $M = 12544$ instead of calculating the entire masks as suggested in [1]. $\mathcal{L}_{mask}$ is a binary mask loss consists of $\mathcal{L}_{ce}$ and $\mathcal{L}_{dice}$ as follows:

$$\mathcal{L}_{mask} = \lambda_{ce}\mathcal{L}_{ce} + \lambda_{dice}\mathcal{L}_{dice}, \quad (1)$$

where $\mathcal{L}_{ce}$ is the binary cross-entropy loss and $\mathcal{L}_{dice}$ is the dice loss. The binary cross-entropy loss $\mathcal{L}_{ce}$ [2] is used to perform mask region classification by considering a bipartite matching-based assignment to compare the predicted value of each mask region with its corresponding ground truth label. The dice loss $\mathcal{L}_{dice}$ [3] aims to optimize the dice overlap coefficient between the predicted segmentation map and the ground truth map so that the predicted segmentation map can properly overlap the ground truth map. The query features generated by the last layer of the transformer decoder, i.e. $\boldsymbol{X}_9$, will go through an MLP layer to generate per-pixel category predictions. The per-pixel category predictions aim to predict possible categories for every pixel of an $H \times W$ image. Here, a classification loss $\mathcal{L}_{cls}$ is computed based on the category labels for every pixel between the prediction and ground truth. The total loss function $\mathcal{L}$ is defined as follows:

$$\mathcal{L} = \mathcal{L}_{mask} + \lambda_{cls}\mathcal{L}_{cls}. \quad (2)$$

B. Implementation Details

It is worth mentioning that the three scale features passed to the transformer decoder in Mask2Former [2] are $1/8$, $1/16$, and $1/32$ of the size of the pathology image. Because our cross-scale guidance integration module already imposes the feature with the largest field-of-view of $1/32$ of the size of the pathology image to guide the features of $1/4$, $1/8$, and $1/16$ of the size of the pathology image, our network can learn the variant shapes and sizes of gland cells on larger scales. By learning gland cells in larger scales, the detailed boundaries can be better decoded by transformer decoders. Thus, the proposed method can have better Hausdorff distance results, as shown in the experiments. Based on observation, our architecture uses the features of $1/4$, $1/8$, and $1/16$ of the size of the pathology image produced by the cross-scale guidance integration module for the transformer decoder instead of $1/8$, $1/16$ and $1/32$ in Mask2Former [2] in the implementation.

TABLE I. Ablation Study for the CRAG Dataset.

Methods	F_1	Dice	Hausdorff
w/o the first block	0.851	0.896	106.45
w/o the second block	0.799	0.863	162.06
Proposed	0.875	0.900	102.11

The proposed method is implemented with the Pytorch 1.9.0 framework with Python 3.8 and runs on a single NVIDIA GeForce RTX 3090 GPU computer. The network is updated using the AdamW optimizer [4]. The initial learning rate is 0.0001, the weight decay is 0.05, and the batch size is 4. In our method, we also use the pre-trained model on the ImageNet [5] dataset due to the small number of pathology images. The parameters of the three losses are λ_{cls} = 2.0, λ_{ce} = 5.0, and λ_{dice} = 5.0, which are suggested in [2]. The source code will be released at https://github.com/nchucvml/CGIT.

II. Results

A. Ablation Study

To assess the impact of the cross-scale guidance integration module, we performed the ablation study on the CRAG dataset [6] and the GlaS dataset [7]. The cross-scale guidance integration module contains two blocks. The first block uses the feature with the largest field-of-view to provide global information for the features with the smaller field-of-views. The second block integrates features of different scales to enrich the multiscale information for the transformer decoder. As shown in Table I and Table II for the CRAG dataset and GlaS dataset, without (w/o) the first block and the second block, all metrics get worse consistently. The reason is that the cross-scale guidance integration module provides more representative cross-scale information for transformer decoders to obtain better segmentation maps. We also show the visualization results on the CRAG dataset in Fig. 1. Pathology images and ground truth labels are shown in Fig. 1(a) and Fig. 1(b), respectively. The results of the proposed method without the first block and the second block are shown in Fig. 1(c) and Fig. 1(d), respectively. Without considering the information for the largest field-of-view and cross-scale information, false alarms and over-segmentation can be observed. In contrast, the proposed method with these two blocks can obtain relatively accurate segmentation boundaries of gland cells, as shown in Fig. 1(e). Fig. 2 shows the visualizations for the GlaS dataset. Similar to the observation in the CRAG dataset, the proposed method can obtain relatively accurate segmentation boundaries of gland cells by considering both blocks, as shown in Fig. 2(e).

TABLE II. Ablation Study for the GlaS Dataset.

Methods	F_1		Dice		Hausdorff	
	TestA	TestB	TestA	TestB	TestA	TestB
w/o the first block	0.894	0.795	0.905	0.830	43.36	107.95
w/o the second block	0.877	0.787	0.895	0.823	49.97	107.47
Proposed	0.919	0.820	0.920	0.851	35.92	87.14

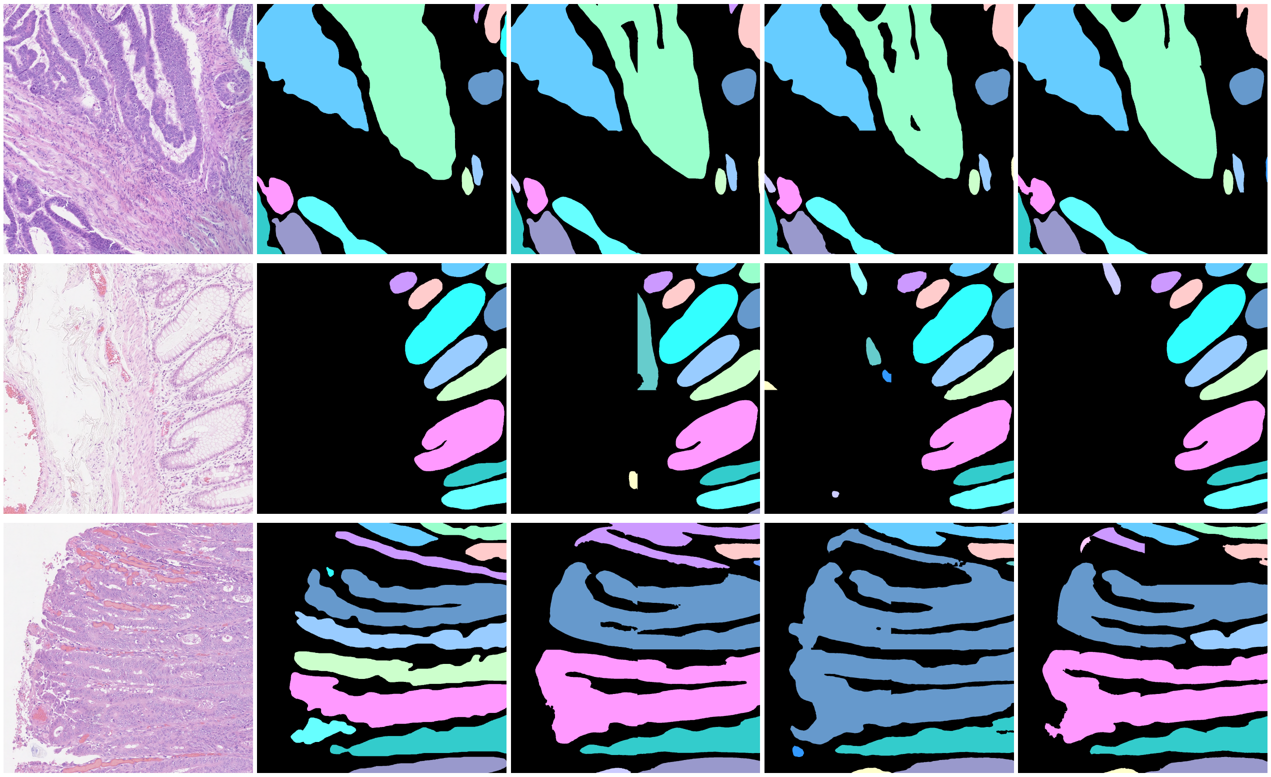

(a) (b) (c) (d) (e)

Fig. 1. Visualizations of the results of the ablation study for the CRAG dataset. (a) Original image, (b) Ground truth, (c) w/o the first block, (d) w/o the second block, and (e) Proposed method.

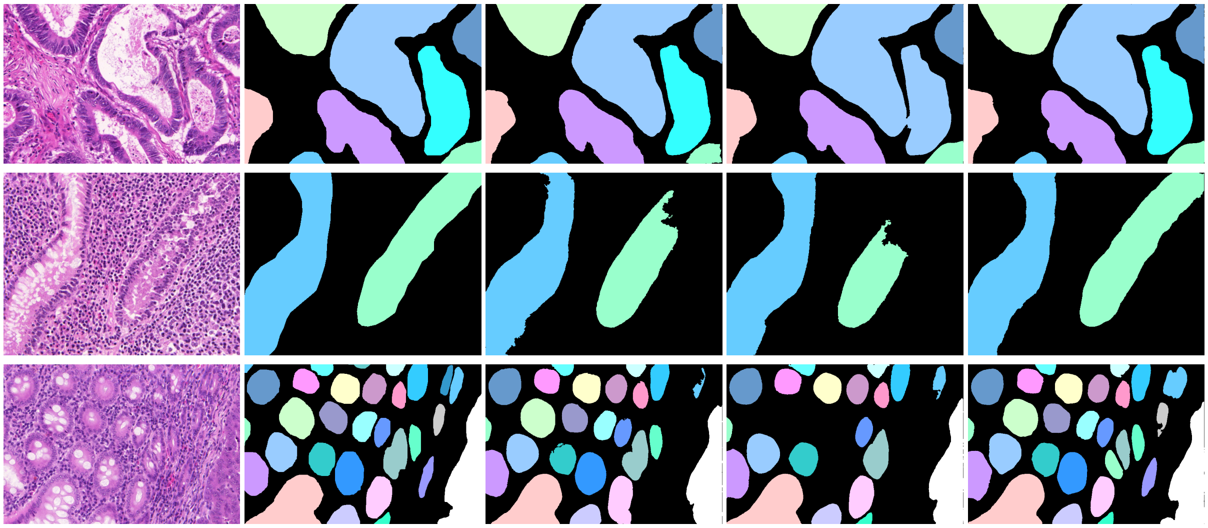

(a) (b) (c) (d) (e)

Fig. 2. Visualizations of ablation study results for the GlaS dataset. (a) Original image, (b) Ground truth, (c) w/o the first block, (d) w/o the second block, and (e) Proposed method.

REFERENCES

[1] A. Kirillov, Y. Wu, K. He, and R. Girshick, “Pointrend: Image segmentation as rendering,” in *Proc. IEEE Conf. Computer Vision and Pattern Recognition*, 2020, pp. 9799–9808.

[2] B. Cheng, I. Misra, A. G. Schwing, A. Kirillov, and R. Girdhar, “Masked-attention mask transformer for universal image segmentation,” in *Proc. IEEE Conf. Computer Vision and Pattern Recognition*, 2022, pp. 1290–1299.

[3] F. Milletari, N. Navab, and S.-A. Ahmadi, “V-net: Fully convolutional neural networks for volumetric medical image segmentation,” in *Proc. Int'l Conf. 3D Vision*, 2016, pp. 565–571.

[4] I. Loshchilov, “Decoupled weight decay regularization,” *arXiv preprint arXiv:1711.05101*, 2017.

[5] J. Deng, W. Dong, R. Socher, L.-J. Li, K. Li, and L. Fei-Fei, “Imagenet: A large-scale hierarchical image database,” in *Proc. IEEE Conf. Computer Vision and Pattern Recognition*, 2009, pp. 248–255.

[6] S. Graham, H. Chen, J. Gamper, Q. Dou, P.-A. Heng, D. Snead, Y. W. Tsang, and N. Rajpoot, “Mild-net: Minimal information loss dilated network for gland instance segmentation in colon histology images,” *Medical Image Analysis*, vol. 52, no. 4, pp. 199–211, 2019.

[7] K. Sirinukunwattana, J. P. Pluim, H. Chen, X. Qi, P.-A. Heng, Y. B. Guo, L. Y. Wang, B. J. Matuszewski, E. Bruni, U. Sanchez *et al.*, “Gland segmentation in colon histology images: The glas challenge contest,” *Medical Image Analysis*, vol. 35, pp. 489–502, 2017.
